# Nanoscale imaging of buried topological defects with quantitative X-ray magnetic microscopy

**DOI:** 10.1038/ncomms9196

**Published:** 2015-09-04

**Authors:** C. Blanco-Roldán, C. Quirós, A. Sorrentino, A. Hierro-Rodríguez, L. M. Álvarez-Prado, R. Valcárcel, M. Duch, N. Torras, J. Esteve, J. I. Martín, M. Vélez, J. M. Alameda, E. Pereiro, S. Ferrer

**Affiliations:** 1 Departamento de Física, Universidad de Oviedo, Oviedo 33007, Spain; 2 Centro de Investigación en Nanomateriales y Nanotecnología, CINN (CSIC—Universidad de Oviedo), El Entrego 33940, Spain; 3 ALBA Synchrotron, Cerdanyola del Vallès 08290, Spain; 4 Departamento de Física e Astronomia, IN-IFIMUP, Universidade do Porto, Porto 4169-007, Portugal; 5 Departamento de Física e Astronomia, INESC-TEC (Coordinated by INESC-Porto), Faculdade de Ciencias, Universidade do Porto, Porto 4169-007, Portugal; 6 Centro Nacional de Microelectrónica, IMB–CNM, CSIC, Campus Universidad Autónoma de Barcelona, Bellaterra 08193, Spain

## Abstract

Advances in nanoscale magnetism increasingly require characterization tools providing detailed descriptions of magnetic configurations. Magnetic transmission X-ray microscopy produces element specific magnetic domain images with nanometric lateral resolution in films up to ∼100 nm thick. Here we present an imaging method using the angular dependence of magnetic contrast in a series of high resolution transmission X-ray microscopy images to obtain quantitative descriptions of the magnetization (canting angles relative to surface normal and sense). This method is applied to 55–120 nm thick ferromagnetic NdCo_5_ layers (canting angles between 65° and 22°), and to a NdCo_5_ film covered with permalloy. Interestingly, permalloy induces a 43° rotation of Co magnetization towards surface normal. Our method allows identifying complex topological defects (merons or ½ skyrmions) in a NdCo_5_ film that are only partially replicated by the permalloy overlayer. These results open possibilities for the characterization of deeply buried magnetic topological defects, nanostructures and devices.

## Abstract (web summary)

Transmission X-ray microscopy allows for the imaging of magnetic domains in thin film materials. Here, the authors exploit the angular dependence of the magnetic contrast to extract out-of-plane canting angles of stripe domains and topological defects in NdCo_5_ films buried under a NiFe layer.

## Graphical abstract

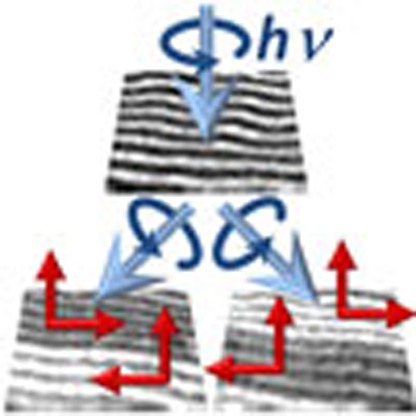

Magnetic domains and singularities are basic ingredients of the microscopic structure of magnetic materials and they exhibit a large variety of morphologies as, to mention some, stripes, mazes, dots, Bloch lines, vortex and skyrmions^1^. These magnetic structures have been brought to evidence by a variety of imaging methods being the most common optical (magnetic Kerr and Faraday effect microscopies), electron-based (Lorentz, spin resolved scattering and photoemission microscopies) or local probes (magnetic force microscopy) and, although they generate good resolution images of the magnetization of the samples (in-plane or perpendicular), they have limited or null capability for quantifying angles, as for example the canting angle with the surface of an inclined magnetization. Transmission X-ray microscopy (TXM) has also been successfully used as imaging technique exploiting the element-specific dichroic-absorption contrast under resonant absorption conditions^2^. Basically, the previous works have been carried out at a fixed geometry between the incoming photon beam and the sample normal^2^,^3^,^4^,^5^,^6^. The most common geometry used is normal incidence to sense the perpendicular magnetization, although off-normal incidence, which allows probing the in-plane magnetization, has been also employed^7^. In this work, we have gone a step further taking advantage of the particularly simple angular dependence of the magnetic contrast on TXM images, which depends on cos*α*, being *α* the angle defined by the direction of the magnetization and that of the X-ray beam^8^. By acquiring series of high resolution images at different α angles, we obtained the angles of the magnetization respect to the surface normal in stripe domains of weak perpendicular magnetic anisotropy (PMA) amorphous NdCo_5_ (denoted as NC) films of different thickness and determined their change when the films were covered with a 40 nm thick permalloy (Py) film. In addition, this method of angle dependent dichroic imaging has allowed us to identify topological defects consisting in 180° rotations of the in-plane magnetization and an out-of-plane reversal (1/2 skyrmions or merons) on a buried NC film that are partially replicated by the Py overlayer. These results may be of practical use in a variety of areas such as: quantitative description of individual topological defects like magnetic vortices^9^,^10^ and skyrmions^11^,^13^,^14^,^15^,^16^,^17^, tilted recording media^18^, design of magnetic heterostructures where the magnetic configuration in each layer has to be determined to understand global properties (such as isothermal exchange bias^19^ and domain replication phenomena^20^,^21^,^22^) and control of topological defect propagation from layer to layer^23^,^24^.

We describe first how to use angular series of magnetic TXM images to quantify the evolution of the magnetization canting angles as a function of film thickness. Then, taking advantage of the high probing depth of the technique, we analyse in detail non-uniform domain patterns in partially reversed layers. As a result, we identify single nanometric meron topological defects and we discuss their role in the magnetization reversal process.

## Results

### Determination of canting angles

**Figure 1:**
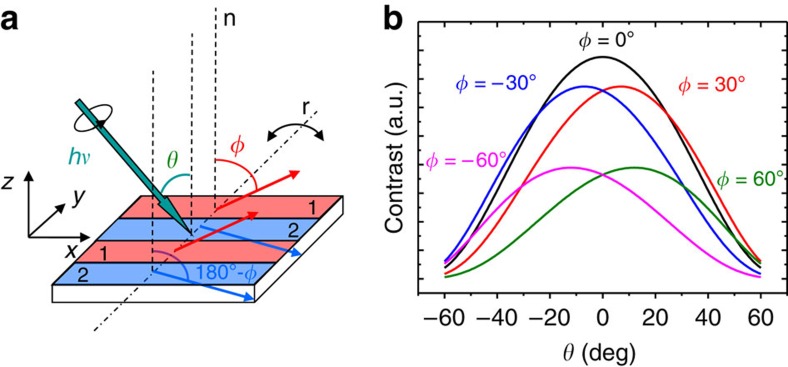
Angular dependent magnetic transmission X-ray microscopy configuration. (**a**) Experimental transmission X-ray microscopy measurement geometry and sketch of the magnetization configuration in a parallel stripe pattern; alternate stripes, denoted as 1 and 2 and shown in red and blue colours, respectively, have opposite *M*_*z*_ components but the same *M*_*x*_, as indicated by the red and blue arrows, that correspond to the magnetization direction in each stripe; *θ* is the angle between the incoming photon beam (indicated by a green arrow and having *hν* energy) and the sample surface normal direction (vertical dotted lines denoted as n); *φ* is the angle between domain 1 magnetization and n direction; sample rotation axis (dashed–dotted line, denoted as r, parallel to the *y* axis and contained in the sample surface plane) allows to vary *θ*. (**b**) Calculated contrast curves *C*(*θ*, *φ*) versus X-ray beam orientation (*θ*) for different domain configurations (*φ* values). For *φ*≠0, the maxima become displaced to positive or negative values depending on the sign of *φ.*

The experimental geometry is described in Fig. 1a. The samples are amorphous NC films with weak PMA^25^. They were prepared to form, at remanence, parallel stripe domain patterns where the out-of-plane component of the magnetization, *M*_*z*_, alternates sign from one stripe to the next (domains 1 and 2 in Fig. 1a), whereas the in-plane component *M*_*x*_ is constant. As NC is a weak PMA material (typical hysteresis loops in Supplementary Fig. 1), the films should exhibit significant variations of the *φ* angles at different thickness (Supplementary Fig. 2 and Supplementary Note 1). In addition, due to topological restrictions^26^,^27^, in-plane magnetization reversal of parallel stripe domains is mediated by the propagation of topological defects (Bloch points) so that NC can be used as a seed layer to nucleate topological defects in a NC/Py bilayer and to investigate their propagation into the soft Py layer.

The samples were mounted with the stripes approximately perpendicular to the rotation axis (*r* in Fig. 1a) of the X-ray microscope of the Mistral beamline at the ALBA light source^28^. The circularly polarized photon beam from a bending magnet source impinged the surface at angles *θ* from *ca*. −55° to +55°. At both kinds of stripe domains (denoted by *i*=1, 2), the magnetization makes angles *φ* and 180° −*φ*, respectively, with the film normal, as indicated in the figure; then, the linear absorption coefficient *μ*_l_^*i*^ of a domain will depend on *L*^−1^(1+*d* **b**(*θ*)˙**m**^*i*^(*φ*)), being *L* the atom and energy dependent X-ray attenuation length, *d* the magnitude of the dichroic absorption, and **b**(*θ*) and **m**^*i*^(*φ*) the unit vectors defining the directions of the beam and magnetization, respectively.

The photon energy was tuned to the *L*_3_ absorption edge of Co. The transmitted intensity of a domain, *I*_*i*_, is governed by exp(−*μ*_l_^*i*^(*θ*,*φ*)*T*/cos*θ*) being *T*/cos*θ* the effective film thickness seen by the X-ray beam. The *θ* dependence of the contrast among both domains *C*(*θ*, *φ*)=|*I*_1_–*I*_2_|, including additional terms to account for the contribution of the attenuation of the beam by Nd atoms and the support membrane, is depicted in Fig. 1b for different values of *φ*.

The calculated curves in Fig. 1b show that the value of *θ* where the contrast is maximum is progressively shifting when |*φ*| increases. Furthermore, if *φ* is positive (negative), as in Fig. 1a, the *θ* dependence of the contrast is centred at positive (negative) angles. Thus, the sign of *φ* provides direct information on the sign of *M*_*x*_, that is, on the orientation of the magnetization.

For a given NC film, images at remanence of a well defined area were acquired at different *θ* angles from *ca.* −55° to +55°, usually in steps of 5°, assuring that the same region of the sample was always imaged. Then, contrast among domains was numerically evaluated and fitted with two adjustable parameters, *φ* and a scale factor, to correlate calculated and measured values.

**Figure 2:**
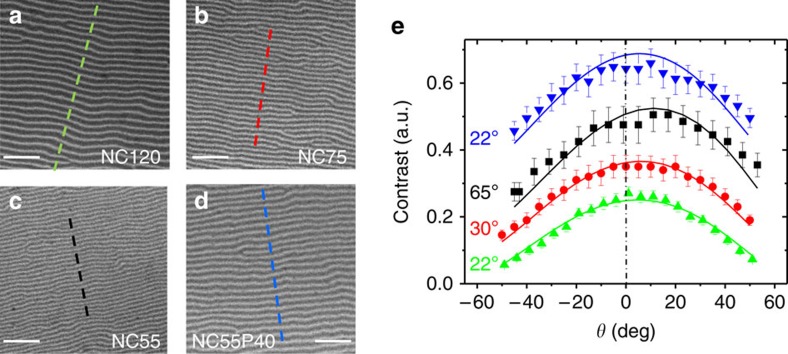
Determination of magnetization canting angles. (**a**–**d**) Normal incidence 4.65 × 4.65 μm^2^ images of NC120, NC75, NC55 and NC55P40 films at remanence, showing the stripe domain pattern typical of systems having perpendicular magnetic anisotropy. Dotted lines indicate the profiles used to evaluate the magnetic contrast. Scale bars, 1 μm. (**e**) Measured (symbols) and calculated (continuous lines) contrasts of the four films (NC120, green up triangles; NC75, red circles; NC55, black squares; NC55P40, blue down triangles) at different angles θ. The fitted values of *φ* are indicated close to the corresponding curves; error bars were evaluated from s.d. of the intensity along the center of the stripes.

Figure 2 shows images of three NC thin films of 55, 75 and 120 nm thickness (denoted as NC55, NC75, and NC120, respectively), and of a 55 nm thickness NC layer covered with 40 nm of Py (denoted as NC55P40). All the images were acquired at the Co absorption edge photon energy and, therefore, they probe the magnetization of Co.

The images in Fig. 2a–d, acquired at *θ*=0°, show well resolved magnetic stripes thanks to the *ca*. 45 nm microscope resolution. The periods of the stripes vary from 187 nm (Fig. 2a, NC120) to 115 nm (Fig. 2c, NC55), in good agreement with previous measurements and micromagnetic calculations^29^. For each sample, a *θ* angular series of images was obtained (the rotation axis was parallel to the vertical direction in the images); then, they were normalized by the flat field images to correct for the background inhomogeneities, and the contrast at each *θ* value was evaluated by averaging the amplitudes of intensity profiles measured along the dashed lines in Fig. 2a–d, that are perpendicular to the stripes. Figure 2e shows the results of the measurements and the calculated curves with the fitted values of the magnetization angle *φ*. Data corresponding to NC120 show the absolute modulation of the contrast with respect to the zero contrast baseline. The other three curves have been vertically shifted for clarity. Typically, the magnetic contrast relative to the total average intensity at normal incidence ranges from 11 to 29%. On decreasing the NC film thickness from 120 to 55 nm, the curves become gradually more asymmetric and the fitted magnetization angle changes continuously from 22° to 65° (the experimental uncertainty of the fitted angles is about ±2°). Referring to Fig. 1a, it corresponds to a decrease of *M*_*z*_ and an increase of *M*_*x*_, that is, magnetization becomes less perpendicular when thickness decreases. This trend is in perfect agreement with micromagnetic simulations (Supplementary Fig. 2 and Supplementary Note 1).

Furthermore, the same procedure applied to the 55 nm NC film buried below 40 nm of Py (upper data and curve in Fig. 2e) indicates a large change in *φ* that decreases back from 65° to 22°; that is, it gets closer to the film normal. This change is accompanied by an increase of the period of the stripes from 115 nm (Fig. 2c) to 179 nm (Fig. 2d). These results again agree with the trend indicated by micromagnetic simulations, which predict a reduction of *φ* correlated to an increment of the period. Thus, the role of the Py layer is to increase the effective thickness of the film, leading to a higher period and to a decrease of *φ*, similarly to the changes observed in uncovered NC films when the thickness is increased from 55 to 120 nm.

### Characterization of topological defects

In a further step, this method was applied to investigate more complex remanent domain patterns in a NC55P40 bilayer after an intermediate state of the in-plane magnetization reversal was generated *ex-situ* by minor hysteresis looping along *x* direction (see Supplementary Fig. 1b). First, a quantitative description of the in-plane domain structure superimposed on oscillating *M*_*z*_-stripe pattern was obtained and then, a detailed characterization of individual topological defects in the magnetization configuration that may nucleate in the system during the magnetization reversal process^26^,^27^ was carried out.

**Figure 3:**
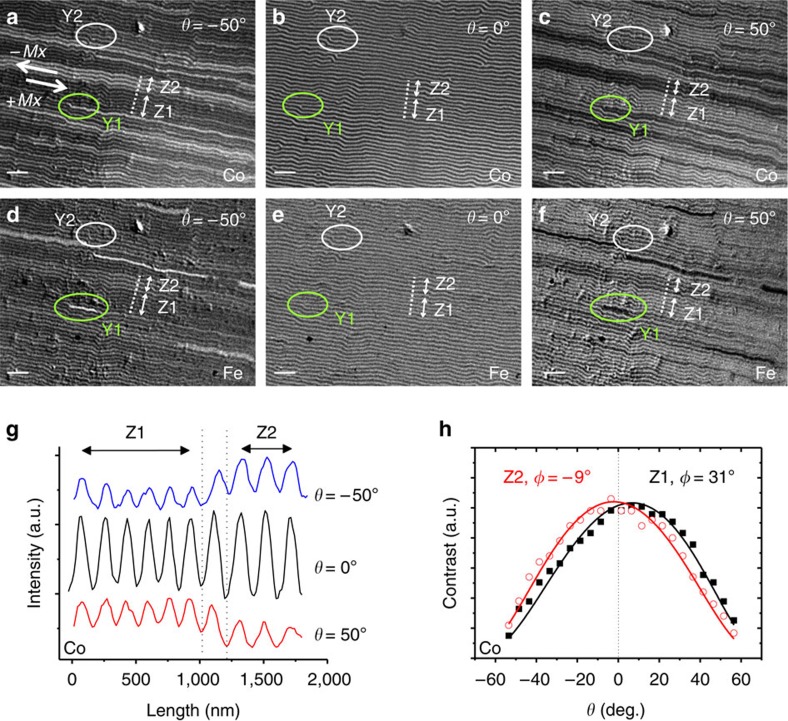
Characterization of in-plane and out-of-plane domain structure in NC/Py bilayer. 12 × 9 μm^2^ magnetic contrast images of the NdCo_5_/Py bilayer: Co magnetization at (**a**) *θ*=−50°, (**b**) *θ*=0° and (**c**) *θ*=+50°, and Fe magnetization at (**d**) *θ*=−50° , (**e**) *θ*=0° and (**f**) *θ*=+50°. Two typical dislocations denoted as Y1 (green ellipses) and Y2 (white ellipses) are shown. Scale bars, 1 μm. (**g**) Intensity profiles (Co edge) across dotted lines in panels (**a**–**c**). (**h**) Angular dependence of contrast curves at Z1 and Z2 regions (fitted *φ*=31° and −9°, respectively); corresponding *M*_*x*_ reversal is indicated by thick arrows in (**a**).

Figure 3 shows two series of images collected at different *θ* angles, either at the Co L_3_ absorption edge photon energy (3a–3c) or at the Fe L_3_ absorption energy (3d–3f), to separately visualize the magnetization of Co (NC layer) and Fe (Py layer). The images were acquired consecutively in the same conditions. At normal incidence (3b and 3e), where the magnetic contrast is only sensitive to *M*_*z*_, both NC and Py layers exhibit identical stripe domains, indicating a replication of the perpendicular magnetization of the NC film to the Py film, similarly to previous findings^20^. However, the images at *θ*=−50° and +50° (Fig. 3a,c,d,f, respectively), that are sensitive to *M*_*x*_, reveal a more complex situation. They are non-homogeneous and exhibit clustering of domains in groups of darker and brighter lines that are not always equivalent in the Co and Fe images.

In more detail, let us concentrate on stripes perpendicular to the dotted line profile in Fig. 3a, acquired at *θ*=−50° for NC. Two different regions, denoted as Z1 and Z2, are observed: in Z1 there is a group of six dark lines with a low average intensity compared with region Z2 that consists of three brighter lines. Both regions are separated by an intermediate contrast transition stripe. At *θ*=+50°, Fig. 3c, the dark/bright sequence is inverted. This inversion is also observed in the Fe images, comparing regions Z1 and Z2 at *θ*=−50° (Fig. 3d), and at *θ*=+50° (Fig. 3f). The similitude of the Co and Fe images indicates that, in this area of the sample, the magnetization pattern of the Py film mimics the magnetization of the NC underneath.

The observed reversal of the contrast as *θ* changes sign indicates that two families of domains coexist with opposite sign of *M*_*x*_. This is confirmed by an analysis of intensity profiles taken across Z1 and Z2 (Fig. 3g) that shows that the amplitudes of oscillations are different in each region. Measuring the specific contrast of Z1 and Z2 in the complete angular series (Fig. 3h) reveals two well separated distributions centred at positive and negative values of *θ*. The continuous lines fitting the data correspond to *φ*=31° for Z1 stripes and −9° for Z2 stripes, confirming a change in sign of *M*_*x*_ as indicated by the two arrows in Fig. 3a. Interestingly, the magnitude of both angles is different. Also, the period of Z2 stripes, ∼195 nm, is slightly larger than that of Z1, ∼173 nm. This confirms the correlation between angle *φ* and period, indicating that the minority reversed stripes have a slightly different energetic balance that affects both period and canting.

**Figure 4:**
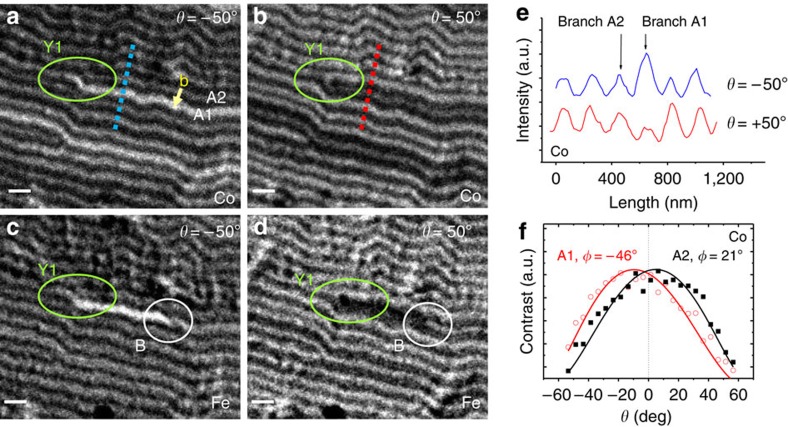
Characterization of an individual dislocation with a meron spin texture at the core. 3.3 × 2.8 μm^2^ magnetic images of NC55P40 around dislocation Y1 (shown in green): (**a**,**b**) Co magnetization at *θ*=−50° and +50°, respectively; note the different contrast between the lower, A1, and upper, A2, branches of dislocation Y1, that indicate *M*_*x*_ reversal starting at the dislocation core; Burgers vector **b** starting at branch A2 and ending at branch A1, is indicated in yellow in panel (**a**). (**c**,**d**) Fe magnetization at *θ*=−50° and +50°, respectively; white circle B highlights a sharp contrast change in branch A1 of Fe images; note the shorter Y1-B segment in (**c**) in comparison with the reversed branch A1 in (**a**). Scale bars, 300 nm. (**e**) Co magnetization intensity profiles across branches A1 and A2 along the dotted lines shown in panels (**a**,**b**). (**f**) Co edge contrast versus *θ* curves corresponding to branches A1 and A2, average canting angles are also indicated.

Finally, after the analysis of the global domain structure, this method has been used to characterize individual magnetization textures, such as dislocations within the stripe domain pattern. These are topological defects that nucleate in the system to adjust stripe period to its equilibrium value, and they are commonly found within magnetic stripe patterns (see for example, Y1 and Y2 in Fig. 3). Normal incidence images (Fig. 3b), sensitive to *M*_*z*_ component, provide two basic dislocation parameters: Burgers vector **b** and polarity change, which are the same for both dislocations Y1 (shown in a closer view in Fig. 4, which is an enlargement of Fig. 3) and Y2. They consist of a white stripe bifurcation (that is, positive *M*_*z*_ domain), next to a black stripe end point with Burgers vector **b** as indicated in Fig. 4a, and of a *M*_*z*_ change from negative to positive on travelling outwards from the dark stripe at the dislocation core to the white stripes that surround it, corresponding to a positive polarity change.

**Figure 5:**
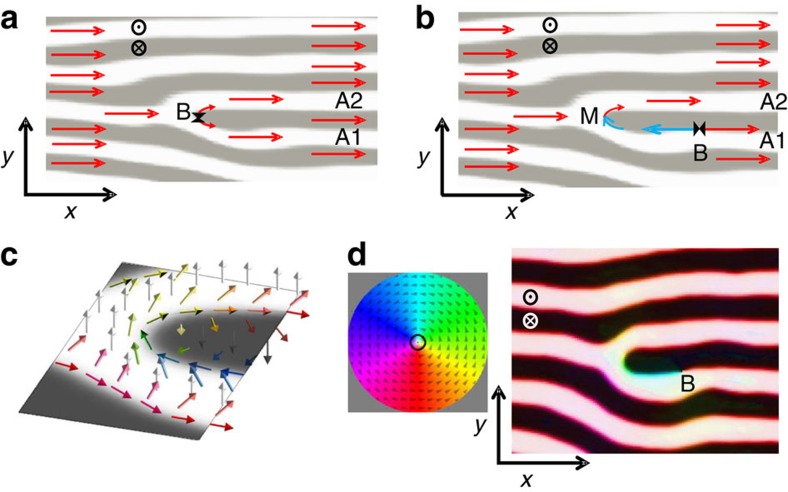
Magnetization configuration at a dislocation core during in-plane magnetization reversal. (**a**) Sketch at remanence with positive *M*_*x*_ (red arrows) in all the stripe pattern, including the dislocation branches A1 and A2. Topological restrictions indicate that a singular Bloch point (B) must appear at the core where the two equally magnetized branches meet tail-to tail along the Bloch wall; *x* and *y* axis, and *z* sense are indicated. (**b**) Sketch with a *M*_*x*_ reversed domain (blue arrows) bounded by a Bloch point (B)—meron (M) pair. (**c**) Three-dimensional (3D) sketch of magnetization (coloured arrows) around a dislocation core with meron. (**d**) 3D micromagnetic simulation of NC layer at central plane showing a Bloch point (B)–meron pair; colour code used to depict the simulated magnetization direction is shown in the coloured wheel.

Moreover, images at different angles evidence additional topological details when in-plane magnetization configuration around the dislocation core is taken into account. In some cases, such as Y2, there are no sharp contrast changes around the dislocation core presenting a similar morphology at *θ*=0° and ±50° images, indicating a uniform in-plane magnetization around the dislocation, as sketched in Fig. 5a. But, in other cases, such as at dislocation Y1, the lower and upper bifurcation branches, denoted as A1 and A2 in Fig. 4a, exhibit different contrasts for Co magnetization (bright-dark, respectively), which are inverted in Fig. 4b at *θ*=+50° (dark-bright), as shown in the profiles in Fig. 4e, indicating a change of sign of *M*_*x*_ at the bifurcation. Again, this is quantitatively confirmed in Fig. 4f by fitting the angular series corresponding to the contrast of each individual branch, A1 and A2, leading to fitted *φ* angles of −46° and 21°, respectively, corroborating the change of sign of *M*_*x*_. This micromagnetic configuration can be described as a meron-like spin texture (M), localized at the dislocation core, that had been predicted to appear at stripe endpoints in helical magnets^30^. As sketched in Fig. 5b,c, the change of sign of *M*_*x*_ at the bifurcation of the white stripe implies a 180° rotation around the end point of the black stripe, that is, there is a ½ turn vortex of the in-plane magnetization plus a polarity change in *M*_*z*_ at the dislocation core. In the particular case of Y1, the meron texture corresponds to a 180° rotation with clockwise chirality and a positive polarity change (Δ*M*_*z*_>0). Linked to this meron texture, we find the narrowest possible in-plane reversed domain, consisting of one single stripe (A1) laterally confined by the *M*_*z*_ stripe periodicity. Contrast changes in Fig. 4 indicate that the magnetization actually performs a single turn helix when crossing branches A2 and A1 along the dotted lines in panels 4a-b, that cross the reversed dislocation core: magnetization changes from positive *M*_*z*_ and positive *M*_*x*_ (white bifurcation branch A2), then to negative *M*_*z*_ (black stripe with end point), then to positive *M*_*z*_ and negative *M*_*x*_ (white bifurcation branch A1), finally back again to positive *M*_*z*_ and positive *M*_*x*_ outside the dislocation core. This single turn helix can be seen in more detail at the right edge of the sketch in Fig. 5c, where, starting from the upper right corner and going down to the lower right corner, the magnetization changes from positive *M*_*z*_ (white arrow), to positive *M*_*x*_ (red arrow), to negative *M*_*z*_ (black arrow), to negative *M*_*x*_ (blue arrow), to positive *M*_*z*_ (white arrow), and back again to positive *M*_*x*_ (red arrow). This configuration is confirmed by the micromagnetic simulation shown in Fig. 5d. Note that we have taken advantage of combined *M*_*x*_-*M*_*z*_ sensitivity of the method to obtain the topological characteristics of individual defects, including chirality and polarity, which are not given by standard characterization techniques, such as Magnetic Force Microscopy, which are sensitive only to *M*_*z*_.

Micromagnetic simulations confirm that these meron structures formed at dislocations play a significant role in the in-plane magnetization reversal process of stripe domain patterns: Fig. 5a shows a sketch of the equilibrium magnetization configuration around a non-reversed dislocation, with a Bloch point^1^ at the bifurcation denoted as B. Simulations indicate that in-plane magnetization reversal starts with a shift of B to the right along the boundary between the black central stripe and one of the white surrounding stripes, A1 in this case. The motion of B leaves a meron configuration at the bifurcation (M) and, between this M-B pair, a *M*_*x*_ reversed domain is formed, (see blue arrows in Fig. 5b,c and the corresponding micromagnetic simulation in Fig. 5d). The A1 reversed stripe experimentally observed in Fig. 4a,b corresponds to a situation where the Bloch point B has moved out of the image.

New details appear when images of Y1 taken at Co edge (Fig. 4a,b) and at the Fe edge (Fig. 4c,d) are compared. Contrast changes are only seen close to the dislocation core at the Fe edge image: a bright (dark) line appears in the segment Y_1_−B for *θ*=−50° (+50°), with a sharp contrast change at point B within the same white stripe. This is consistent with an abrupt *M*_*x*_ change (either a Bloch point or a vertical Bloch line^1^), as indicated in sketch Fig. 5b and simulation Fig. 5d. In fact, several of these short domains bounded by Bloch line-meron pairs can be identified within the full view image of the Py layer in Fig. 3d,f, which have a smaller length than the corresponding reversed domains within the NC layer (Fig. 3a,c). The fact that these narrow in-plane reversed domains are shorter in Py than in NC indicates that their energy per unit length has to be relatively high since the Py film prefers to have short domains on top of long domains in the NC film even at the expense of non-parallel coupling between Py and NC.

Our results illustrate that X-ray microscopy complements other techniques of domain visualization. Its lateral resolution, ∼45 nm in our case, is intermediate between electron-based techniques, as photoemission microscopy or electron diffraction, and optical techniques as Kerr microscopy, and it might be further improved by a factor ∼2 upgrading the objective optics of the microscope. The ability of obtaining canting angles and details on the magnetization of magnetic singularities, as shown in this report, compares with what it is achievable with low energy electron microscopy as demonstrated in recent works^31^,^32^ where in-plane orientations of the magnetization in the domain walls at the surface were determined in perpendicularly magnetized films. Other recent works are also reporting steps towards three-dimensional X-ray magnetic imaging with photoemission electron microscopy^33^ and TXM^34^. However, a specific and rather unique aspect of TXM is its relatively large probing depth coupled to its atomic specificity. Electron-based or optical techniques are inherently surface sensitive (few nm of probing depth for electrons, and few tens of nm for optical wavelengths in non-transparent materials) whereas TXM allows to probe films of more than 100 nm (Fig. 2). As the depth of focus of the microscope is about 6 μm (for the focusing optics and energies used in this experiment) and the intensity analysis is performed close to the centre of rotation, the whole film is always focused in the images and the sharpness of the magnetic features does not depend on their depth. This makes TXM suitable for observation of domains and characterization of chirality and polarity of topological defects in the bulk of non-transparent materials, which is virtually impossible with other techniques^1^. Furthermore, we plan to generalize our method to be applicable to complex domain morphologies, including non-periodic patterns, by precisely measuring the absorption coefficient of the samples, within the lateral resolution, in order to directly obtain a mapping of the *M*_*x*_ and *M*_*z*_ projections of the magnetization. This, combined with the usage of two orthogonal rotation axes, so that the *M*_*y*_ component can also be extracted, is expected to become a powerful tool for magnetic domain imaging.

In summary, we have shown that the simple dependence of magnetic dichroic contrast in angular series of magnetic TXM images can be successfully used as a method to quantify magnetization canting angles, even in deeply buried layers. Film thickness dependence of canting angles obtained by this approach is in agreement with trends predicted by micromagnetic calculations. Furthermore, the application of this analysis to a partially reversed magnetic bilayer has allowed the identification of groups of stripes with opposite *M*_*x*_ sign having different values of their canting angle, in correlation with changes in stripe domain periodicity. Finally, the technique has been used to quantify canting angles in individual 90–100 nm wide stripes around dislocation defects, and meron textures with a defined chirality have been identified. These topological defects, that are not completely replicated to the permalloy overlayer, play a significant role in the magnetization reversal process. In the near future we plan to enlarge the capabilities and generality of the method to probe the three dimensional magnetization of more complex systems with domain configurations of practical interest.

## Methods

### Sample preparation

Amorphous NC films were prepared at room temperature by DC magnetron co-sputtering of Nd (99.9 % atomic purity) and Co (99.99 % atomic purity) targets at 3 × 10^−3^ mbar Ar working pressure. Their thicknesses were varied between 55 and 120 nm. An additional 5 nm thick Al capping layer was grown on top of the samples to prevent oxidation. The saturation magnetization and PMA of these films are^25^,^29^, respectively, *M*_S_=10^3^ e.m.u. cm^−3^ and *K*_N_=10^6^ erg cm^−3^, and therefore, anisotropy ratio *Q*=*K*_N_/2*πM*_S_^2^ is <1. The remanent domain configuration, obtained by cycling an in-plane magnetic field up to 4 kOe from positive to negative magnetic saturation (Supplementary Fig. 1), consists of parallel stripe domains oriented along the last saturating field direction. The canting angle of the stripe pattern *φ* in this low *Q* material is expected to show large variations as a function of thickness (Supplementary Fig. 2), what makes it an ideal candidate to test the validity of our method.

A bilayer sample, consisting of 40 nm of Py directly deposited on top of 55 nm of NC, was also prepared by DC magnetron sputtering from a Py target in similar experimental conditions as those described above.

All of the films were grown on specially designed substrates consisting on 100 nm thick Si_3_N_4_ membranes fabricated by Microsystems technology at IMB–CNM CSIC (Barcelona, Spain). The shape of the Si frame was optimized to maximize the photon beam exit angles, so that a wide angular range of *ca*. ±60° around substrate normal is accessible to acquire the angular series of images without shadowing of the beam.

### Transmission soft X-ray microscope

The images were acquired with the full field transmission soft X-ray microscope of the MISTRAL beamline at the ALBA synchrotron facility (Barcelona, Spain)^28^. The monochromatic X-ray beam was focused with a glass capillary at the sample that was mounted on a high precision rotary axis (<0.5 μm run out). The circular polarization rate was experimentally estimated as 96% (ref. 35). The transmitted beam was magnified to a charge-coupled device chip with a Fresnel zone plate of 40 nm outer zone width (depth of focus of around 6 μm at our experimental conditions). The resolution of the microscope, operating with circularly polarized photons, is about 30 nm half-pitch, as determined with reference calibrated lithographic patterns by acquiring transmission images based on absorption contrast due to different charge densities. However, for magnetic images the resolution degrades due to the skewed illumination inherent to the selection of the circular polarization. An estimation from the visibility of the magnetic contrast in a sample with ∼120 nm period magnetic stripes was *ca*. 45 nm half-pitch. Series of angular images were acquired automatically with exposition times around 10–20 s depending on the angles and on the sample thickness. For large incoming angles exposition times were increased to keep similar signal-to-noise ratios. The background was corrected normalizing to a flat field image obtained without any sample and, subsequently, the images were aligned to correct for the run out error of the rotation axis to compare exactly the same areas of the sample at different angles. Separate X-ray absorption measurements^36^ allowed to determine *L*=22 nm and *d*=0.27 in NC in agreement with literature^8^. Error bars in Fig. 2e were evaluated from s.d. of the intensity along the centre of the stripes.

### Micromagnetic simulations

Average canting angles of regular stripe patterns at remanence were obtained from micromagnetic simulations with a finite difference code^37^ in which the continuous thin films were modelled as infinite prisms along the stripe pattern direction and two dimensional rectangular cross-section discretized as (thickness/64) × (period/64) nm^2^. Finite-element code^38^ MuMax3 was used to simulate the magnetization reversal of three-dimensional NC films to allow for the nucleation of dislocation in the stripe pattern. The films were discretized into cells with dimensions of 4 × 4 × 2 nm^3^ for a total of 1,024 × 1,024 × 56 nm^3^. Material parameters have been obtained from the magnetic characterization of the samples (*M*_S_≈10^3^ e.m.u. cm^−3^, *K*_N_≈10^6^ erg cm^−3^) and exchange constant *A*_ex_≈4 × 10^−7^ erg cm^−1^.

## Additional information

**How to cite this article:** Blanco-Roldán, C. *et al*. Nanoscale imaging of buried topological defects with quantitative X-ray magnetic microscopy. *Nat. Commun.* 6:8196 doi: 10.1038/ncomms9196 (2015).

## Supplementary Material

Supplementary Information Supplementary Figures 1-2, Supplementary Note 1 and Supplementary Reference
