# Supplementary material for: Nanoscale imaging of buried topological defects with quantitative X-ray magnetic microscopy
Source: Nat Commun. 2015 Sep 4;6:8196. doi: 10.1038/ncomms9196 (PMC4569793; doi:10.1038/ncomms9196)
Supplement: Supplementary Information — Supplementary Figures 1-2, Supplementary Note 1 and Supplementary Reference [file ncomms9196-s1.pdf]

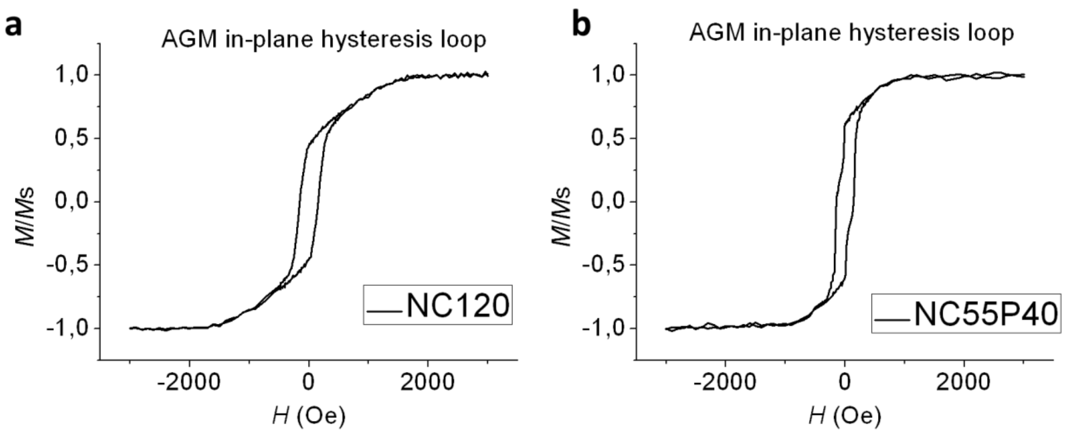


**Supplementary Figure 1 | Experimental in-plane hysteresis cycles.** (**a**) In-plane magnetic hysteresis loop of NC120 nm normalized to saturation magnetization measured by Alternating Gradient Magnetometry (AGM). It has the characteristic shape of in-plane transcritical loops corresponding to weak perpendicular magnetic anisotropy (PMA) films. (**b**) In-plane AGM hysteresis loop of bilayer NC55P40. Minor loops were performed in this sample in order to get a partially reversed magnetic state.

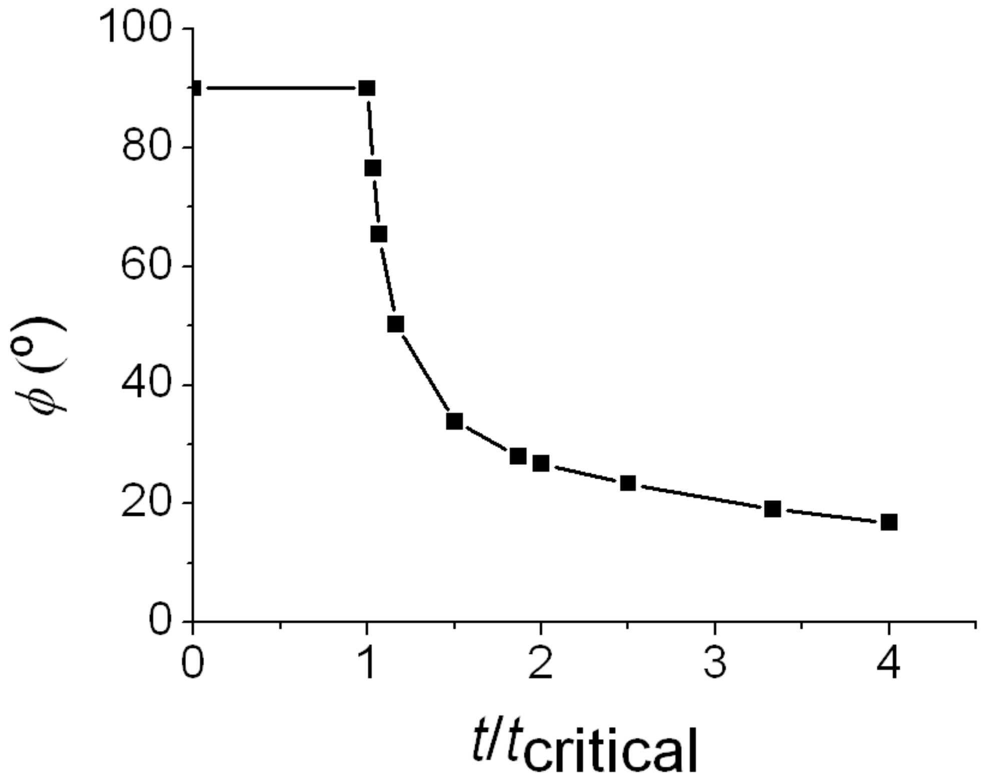


**Supplementary Figure 2 | Calculated dependence of magnetization canting angle $\phi$ with film thickness.** Canting angles at remanence obtained from micromagnetic simulations as a function of film thickness normalized to $t_{critical}$, that is, the value of the thickness corresponding to the in-plane to out-of-plane transition. The results indicate that there is a critical thickness below which the easy axis is in the film plane, whereas above it, a stripe domain pattern is formed with a canting angle continuously decreasing with thickness, so that the film magnetization gets progressively more perpendicular. Close to the critical thickness $\phi$ is maximum and varies very rapidly. Away from the transition, this variation slows down approaching values around 20º, in good agreement with our experimental findings. Additional discussion is given in Supplementary Note 1.

**Supplementary Note 1. Micromagnetic simulations**.

The calculated canting angle $\phi$ at remanence as a function of film thickness was found by simulating the part of the hysteresis loop which starts around the saturation state and finishes at zero magnetic field. The hysteresis loop was numerically obtained by lowering the external magnetic field in a series of successive steps. For each magnetic field step the domains evolved until an equilibrium configuration was reached. As the stripe period depends on the magnetic field, an optimized period has to be found at a given magnetic field. This involves finding the minimum energy among several stripe periods for the magnetic field considered[1]. The outputs of the 2-D micromagnetic simulations are periodic bidimensional distributions of the canting angle inside the magnetic thin film $\phi$ $(y,z)$. In order to compare these results with the measured X-ray transmission images, the thickness-average of $\phi$ (at the center of each stripe) around its maximal value in the direction perpendicular to the stripes has been calculated. This average is the $\phi$ value represented in Supplementary Figure 2.

**Supplementary Reference**


1 B. B. Pant and K. Matsuyama, Jap. J. Appl. Phys. **32** (1993) 3817-3822
